# Isolated Pericardial Hydatid Cyst: A Case Report

**DOI:** 10.4021/cr96w

**Published:** 2011-09-20

**Authors:** Hasan Cece, Sema Yildiz, Ozgur Sogut, Abdussamet Hazar, Yusuf Sezen

**Affiliations:** a Harran University, Faculty of Medicine, Department of Radiology, Sanliurfa, Turkey; b Harran University, Faculty of Medicine, Department of Emergency Medicine, Sanliurfa, Turkey; c Harran University, Faculty of Medicine, Department of Cardiovascular Surgery, Sanliurfa, Turkey; d Harran University, Faculty of Medicine, Department of Cardiology, Sanliurfa, Turkey

**Keywords:** Echinococcosis, Cardiac hydatid disease, Pericardial hydatid cyst, Computed tomography, Magnetic resonance imaging

## Abstract

Cardiac echinococcosis is extremely rare, and cysts are found mostly within the myocardium. Most cardiac hydatid cysts are located in the left ventricular wall. Only a few cases of isolated pericardial hydatid cysts have been reported. Echocardiography, computed tomography and magnetic resonance imaging are important diagnostic tools for the diagnosis of echinococcosis. Herein, we report a rare case of isolated pericardial hydatid cyst who presented to our emergency department with complaints of mid-sternal chest pain and shortness of breath.

## Introduction

Hydatid cysts caused by *Echinococcus granulosus* are usually located in the liver and lungs; cardiac involvement is quite rare. The incidence of cardiac echinococcosis is <2% of the total echinococcosis incidence, even in countries endemic for echinococcosis [1]. The anatomical site of location in the heart most commonly includes the left ventricle; cysts are rarely located in the right ventricle, interventricular septum, and atrium [2]. Isolated pericardial hydatid cysts are quite rare [3, 4]. Herein, we present computed tomography (CT) and magnetic resonance imaging (MRI) findings of a patient with a hydatid cyst located in the pericardium adjacent to the left ventricle, together with a review of the literature.

## Case Report

**Figure 1:**
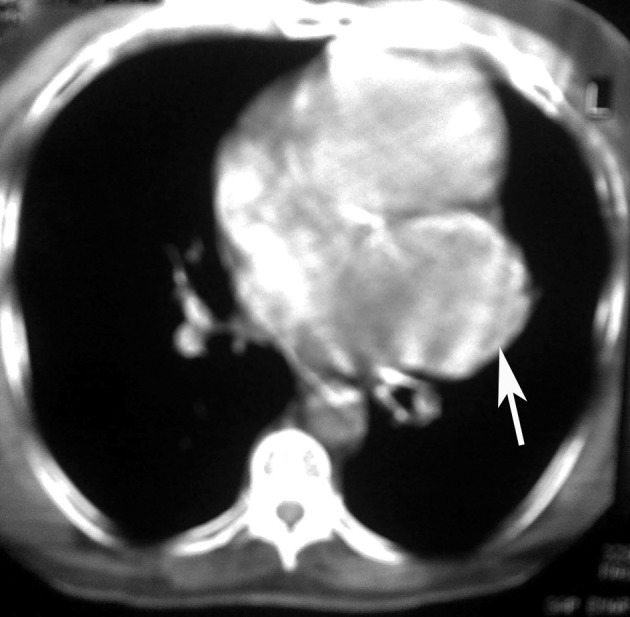
Axial CT scan of the thorax showing solitary unilocular cystic lesion on the left side of pericardium (white arrow).

**Figure 2:**
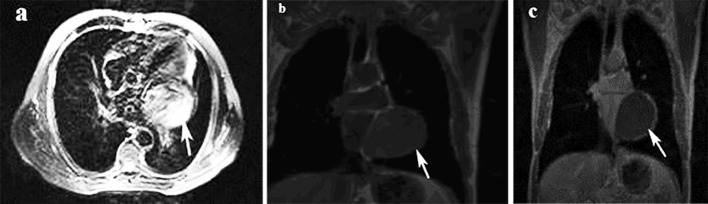
Axial T2 weighted MR image (a), coronal T1 weighted MR image (b) and contrast-enhanced coronal T1 weighted MR image (c) show high signal intensity content on T2, low signal intensity content on T1 and non enhanced on contrast-enhanced T1 solitary unilocular cystic lesion on the left side of pericardium (white arrows).

A 47-year-old female patient presented to our emergency department with complaints of shortness of breath and chest pain. Her medical history was unremarkable. On her physical examination, arterial blood pressure was 125/70 mmHg, heart rate was 86 beats per minute, and respiratory rate was 16 breaths per minute. Cardiac and pulmonary auscultation was normal. Cardiac enzymes, biochemical analysis, and complete blood count were normal except for eosinophilia. Posterior-anterior chest radiograph showed normal pulmonary appearance; however, a hyperdense mass with regular contours, measuring 5 cm in diameter, was detected in the apical aspect of the left ventricle. Electrocardiographic (ECG) findings were unremarkable. A transthoracic echocardiogram (TTE) showed a cystic mass lesion located in the pericardium adjacent to the left ventricle. Thoracic CT and MRI were performed for differential diagnosis of the cardiac mass. Her CT showed a 5 x 6 cm cystic lesion with well-defined borders in the pericardium adjacent to the left ventricle (Fig. 1). On MRI, a cystic lesion adjacent to the posterior aspect of the left ventricle was observed; the lesion was hyperintense on axial T2-weighted images, hypointense on axial and coronal T1-weighted images, and did not show contrast enhancement on postcontrast coronal T1-weighted images (Fig. 2). The diagnosis was verified by an indirect hemagglutination test performed to the patient who had been considered to have a cardiac hydatid cyst. Abdominal ultrasonographic evaluation revealed no other cystic lesion either in the liver or other abdominal organs. Under the light of these findings, the patient was scheduled for surgery with the diagnosis of cardiac hydatid cyst. The mediastinum was accessed through left anterolateral thoracotomy. Accessing to the left mediastinal area, a solitary cystic lesion with a well-defined border was visualized in the pericardium adjacent to the left ventricle. The lesion was aspirated via puncture. Because the cyst was adhered to the myocardial wall and surrounding tissue, it was resected leaving no potential space as possible. Pathologic examination of the specimen was consistent with hydatid cyst. The patient had no problem in the postoperative period. Albendazole was commenced at a dose of 10 mg/kg/day for three months for the patient that developed no complications during the postoperative period. The patient was discharged on the eighth day. Neither a problem nor a hydatid cyst relapse was noted during her 2-year follow-up period.

## Discussion

Pericarditis may occur as a result of spontaneous rupture or secondary infections of superficial cardiac or pericardial hydatid cysts. Moreover, cyst rupture in the ventricle wall may cause constrictive pericarditis. Establishing the diagnosis and applying the treatment before the development of complications, are life-savings in cases with cardiac hydatid cysts, which are rarely encountered. The embryos, which reach the right atrium from the intestine through the liver and azygos vein, access into the left ventricle and coronary arteries. Scolices may reach the heart by following the same way as mature hepatic cysts, or via the rupture of a lung cyst into the pulmonary vein [1]. Nearly, 0.02%-2% of the hydatid cysts is located in the heart. The anatomical site of location in the heart depends on the amount of regional vascularization. It has been reported that, 50%-60% of the cardiac hydatid cysts are located in the left ventricle, 10%-20% are located in the interventricular septum, 5%-15% are located in the right ventricle, 10%-15% are located in the pericardium, and 5%-8% are located in the right or left atrium [2]. In their series involving 60 cases, Akar *et al*. [5] found the left ventricle to be the most common site of location by 47%, followed by the right ventricle (19%), interventricular septum (19%) and right atrium (11%). Isolated pericardial hydatid cyst has been reported as a rare entity [3, 4]. During an 18-year period, Salati *et al*. [3] found isolated pericardial hydatid cysts in only 11 (1.4%) of 783 cases of cardiothoracic hydatid cysts. In the present case, the hydatid cyst was located in the pericardium adjacent to the left ventricle.

Patients with cardiac hydatid cysts most commonly present to the emergency department with dyspnea, palpitation, and chest pain [5, 6]. Other symptoms may include fever, hemoptysis, cough, anaphylactic shock, syncope, arrhythmia and conduction disturbances, acute myocardial infarction, precordial pain, valvular dysfunction, pulmonary hypertension, pulmonary and systemic embolism, hemiplegia, and loss of consciousness. Complications may cause sudden death [5-7]. The clinical symptoms of the present case were chest pain and dyspnea. Nevertheless, it should be kept in mind that patients with cardiac hydatid cyst may be asymptomatic [5]. As soon as the diagnosis is established, surgical excision should be performed immediately for potential complications even if the patient is asymptomatic [8, 9].

Although there is no characteristic ECG findings of a hydatid cyst, nonspecific repolarization changes, atrioventricular and intraventricular block, and complex premature ventricular contractions are likely to be observed [7, 10].

Diagnostic methods for cardiac hydatid cysts include serological tests, as well as noninvasive imaging techniques such as TTE, CT and MRI [11]. TTE is in the forefront because it is widely available, easy to use and is highly sensitive in the diagnosis of intracardiac cysts [2, 6]. Although cardiac hydatid cysts are rarely seen, some authors recommend routine TTE screening in cases with hepatic or pulmonary hydatid cysts [12, 13]. Following TTE, CT is the second diagnostic method of choice in cases with cardiac hydatid cysts, and CT shows wall calcification well [11, 14]. However, obtaining sections in a single, axial plane may appear as a disadvantage and may cause small lesions to be missed [15]. Cardiac motion artifact as well is an important limitation of CT. On the other hand, MRI allows multiplanar investigation of cardiac cysts and their contents. Cardiac-gated MRI is complementary and provides more detailed information than CT, particularly in cases with cardiac echinococcosis where TE remains incapable [2]. Images obtained using the cine gradient echo technique not only provide morphological information about cardiac cysts, but also display the effect of cysts on cardiac functions [16].

In conclusion, although cardiac hydatid cyst is a rare disease, early diagnosis and treatment is of great importance because of frequent fatal complications. TTE, followed by CT and MRI are important diagnostic methods. Surgical excision is the best treatment modality for cardiac hydatid cysts located in the pericardium.

## References

[1] Perez-Gomez F, Duran H, Tamames S, Perrote JL, Blanes A (1973). Cardiac echinococcosis: clinical picture and complications. Br Heart J.

[2] Polat P, Kantarci M, Alper F, Suma S, Koruyucu MB, Okur A (2003). Hydatid disease from head to toe. Radiographics.

[3] Salati SA, Dar AM, Khan AB, Bhat MA, Ahangar AG (2008). Isolated pericardial hydatid cyst. J Surg Pak.

[4] Vural M, Sayin B, Pasaoglu L, Koparal S, Elverici E, Dede D (2007). Isolated pericardial hydatid cyst in an asymptomatic patient: a remark on its radiologic diagnosis. Clin Imaging.

[5] Akar R, Eryilmaz S, Yazicioglu L, Eren NT, Durdu S, Uysalel A, Ucanok K (2003). Surgery for cardiac hydatid disease: an Anatolian experience. Anadolu Kardiyol Derg.

[6] Ben-Hamda K, Maatouk F, Ben-Farhat M, Betbout F, Gamra H, Addad F, Fatima A (2003). Eighteen-year experience with echinococcosus of the heart: clinical and echocardiographic features in 14 patients. Int J Cardiol.

[7] Molavipour A, Javan H, Moghaddam AA, Dastani M, Abbasi M, Ghahramani S (2010). Combined medical and surgical treatment of intracardiac hydatid cysts in 11 patients. J Card Surg.

[8] Gurbuz A, Tetik O, Yilik L, Emrecan B, Ozsoyler I, Ozbek C (2003). Cardiac involvement of hydatid disease. Jpn J Thorac Cardiovasc Surg.

[9] Murat V, Qian Z, Guo S, Qiao J (2007). Cardiac and pericardial echinococcosis: report of 15 cases. Asian Cardiovasc Thorac Ann.

[10] Yuksel S, Gulel O, Elmali M, Kale A, Sahin M (2009). Huge cardiac cyst hydatid causing cardiac symptoms and electrocardiographic changes. Eur J Echocardiogr.

[11] Dursun M, Terzibasioglu E, Yilmaz R, Cekrezi B, Olgar S, Nisli K, Tunaci A (2008). Cardiac hydatid disease: CT and MRI findings. AJR Am J Roentgenol.

[12] Bashour TT, Alali AR, Mason DT, Saalouke M (1996). Echinococcosis of the heart: clinical and echocardiographic features in 19 patients. Am Heart J.

[13] Tuncer E, Tas SG, Mataraci I, Tuncer A, Donmez AA, Aksut M, Yakut C (2010). Surgical treatment of cardiac hydatid disease in 13 patients. Tex Heart Inst J.

[14] Gouliamos AD, Kalovidouris A, Papailiou J, Vlahos L, Papavasiliou C (1991). CT appearance of pulmonary hydatid disease. Chest.

[15] Siwach SB, Katyal VK, Jagdish (1997). Cardiac echinococcosis—a rare echocardiographic diagnosis. Heart.

[16] von Sinner W, te Strake L, Clark D, Sharif H (1991). MR imaging in hydatid disease. AJR Am J Roentgenol.

